# Atypical rearrangement involving 3′-*IGH@* and a breakpoint at least 400 Kb upstream of an intact *MYC* in a CLL patient with an apparently balanced t(8;14)(q24.1;q32) and negative *MYC* expression

**DOI:** 10.1186/1755-8166-6-5

**Published:** 2013-02-01

**Authors:** Ina Amarillo, Peter H Bui, Sibel Kantarci, Nagesh Rao, Brit S Shackley, Rolando García, Carlos A Tirado

**Affiliations:** 1 Clinical Molecular Cytogenetics Laboratory, Medicine, David Geffen UCLA School of Medicine, Los Angeles, CA, USA; 2 Department of Pathology & Laboratory, Medicine, David Geffen UCLA School of Medicine, Los Angeles, CA, USA; 3 Cytogenetics, UT Southwestern Medical Center, Dallas, TX, USA

**Keywords:** MYC/IGH, FISH, CLL, Microarray

## Abstract

The t(8;14)(q24.1;q32), the cytogenetic hallmark of Burkitt’s lymphoma, is also found, but rarely, in cases of chronic lymphocytic leukemia (CLL). Such translocation typically results in a *MYC*-*IGH@* fusion subsequently deregulating and overexpressing *MYC* on der 14q32. In CLL, atypical rearrangements resulting in its gain or loss, within or outside of *IGH@* or *MYC* locus, have been reported, but their clinical significance remains uncertain. Herein, we report a 67 year-old male with complex cytogenetic findings of apparently balanced t(8;14) and unreported complex rearrangements of *IGH@* and *MYC* loci. His clinical, morphological and immunophenotypic features were consistent with the diagnosis of CLL.

Interphase FISH studies revealed deletions of 11q22.3 and 13q14.3, and an extra copy of *IGH@,* indicative of rearrangement. Karyotype analysis showed an apparently balanced t(8;14)(q24.1;q32). Sequential GPG-metaphase FISH studies revealed abnormal signal patterns: rearrangement of IGH break apart probe with the 5’-IGH@ on derivative 8q24.1 and the 3’-IGH@ retained on der 14q; absence of MYC break apart-specific signal on der 8q; and, the presence of unsplit 5’-*MYC*-3’ break apart probe signals on der 14q. The breakpoint on 8q24.1 was found to be at least 400 Kb upstream of 5’ of *MYC*. In addition, FISH studies revealed two abnormal clones; one with 13q14.3 deletion, and the other, with concurrent 11q deletion and atypical rearrangements. Chromosome microarray analysis (CMA) detected a 7.1 Mb deletion on 11q22.3-q23.3 including *ATM*, a finding consistent with FISH results. While no significant copy number gain or loss observed on chromosomes 8, 12 and 13, a 455 Kb microdeletion of uncertain clinical significance was detected on 14q32.33. Immunohistochemistry showed co-expression of CD19, CD5, and CD23, positive ZAP-70 expression and absence of *MYC* expression. Overall findings reveal an apparently balanced t(8;14) and atypical complex rearrangements involving 3’-*IGH@* and a breakpoint at least 400 Kb upstream of *MYC*, resulting in the relocation of the intact 5’-*MYC*-3’ from der 8q, and apposition to 3’-*IGH@* at der 14q. This case report provides unique and additional cytogenetic data that may be of clinical significance in such a rare finding in CLL. It also highlights the utility of conventional and sequential metaphase FISH in understanding complex chromosome anomalies and their association with other clinical findings in patients with CLL. To the best of our knowledge, this is the first CLL reported case with such an atypical rearrangement in a patient with a negative *MYC* expression.

## Background

Chronic lymphocyctic leukemia (CLL) is the most common leukemia in the elderly with clinical presentation of lymphocytosis, bone marrow involvement, lymphadenopathy, hepatosplenomegaly, complex cytogenetics and heterogeneous clinical course [1]. Immunophenotypically, aberrant expression of CD5, CD20, CD22, CD23, CD38, CD43 and CD79 is diagnostic or prognostic of B-cells in CLL [2]. Common cytogenetic anomalies include deletion of 13q14.3 (most frequent) and/or 13q34, deletion of 11q, deletion of 17p, trisomy 12 and *IGH@* rearrangement [3].

While t(8;14)(q24.1;q32), the cytogenetic hallmark of Burkitt’s lymphoma, is a primary genetic event found in about 70-80% of cases, it is usually a rare secondary anomaly in other B-cell disorders including CLL (about 0.2% to <1%) [4-8], lymphoblastic leukemia, DLBCL, and in other lymphoma transforming into a more aggressive disease [9]. In the latter, t(8;14) usually confers favorable prognosis, while a more aggressive phenotype and poor outcome are manifested when it is a part of a complex chromosome complement [5,10].

In a typical t(8;14)(q24;q32) translocation, the *MYC* at 8q24.1 locus is spatiotemporally juxtaposed with the 3’-*IGH@* locus on derivative 14q32 [11-15]. The *IGH* transcription factory, about 2.5 Mb in size [12], localizes the regulatory elements for *MYC* deregulation and variable regions that promote translocation [13]. The *IGH@* locus, is a hotspot for recombination and mutation of immunoglobulin genes during B-cell maturation, processes that usually promote translocations with oncogenic potential [11]. Whereas the breakpoint on chromosome 14 is within the *IGH@* locus, usually located within the μ-gene, either within or adjacent to the variable (V), joining (J), diversity (D, or switch (S) regions, but other heavy-chain regions are occasionally involved [9]. While about 80% of translocations in Burkitt’s lymphoma is typical and involve *MYC* and *IGH@* (IG heavy chain) [16], others are involved in variant partnership with other IG chain loci; kappa light chain (*IGK*) at 2p12, or lambda light chain (*IGL*) at 22q11.2 [16-18]. *MYC* is also involved with *IGH* in DLBCL [18], *TCR* alpha/delta in T-acute lymphoblastic leukemia/lymphoma, and IG kappa and lambda chains in plasma cell myeloma [18,19].

*MYC* is a proto-oncogene that encodes for a transcription factor that regulates cell cycle progression, growth, differentiation, apoptosis, survival and biosynthesis [4,6,20]. It activates or represses transcription factories of other genes (about 10%), transcription factors, and chromatin modifying and remodeling complexes [20]. Rearrangements involving *MYC* drive cells into lymphomagenesis often through its deregulation and overexpression [5,11,12,21,22]. The oncogenic potential of *MYC* rearrangements is implicated not only in the initiation of lymphomagenesis but also in its transformation and progression of low-grade lymphomas into a more advanced disease and an unfavorable outcome [5,17,18,21-23]. These findings suggest that the level of deregulated *MYC* expression of different stages of aberrant cellular maturation and differentiation may influence the neoplastic phenotype [9].

At 8q24.1 locus, translocation breakpoints are located within or surrounding the *MYC*: regulatory region within *MYC*, from exon 1 to intron 1, (Class I and most common); transcription factor binding-site at or adjacent to 5’-*MYC* (Class II); and long-range regions up to 100-300 Kb or more upstream from an intact 5’-*MYC*-3’ (Class III) [15,16,20]. It has been suggested that aberrant *MYC* expression is influenced by breakpoint location, mutation within the translocated region, deletion of regulatory elements, or transcription at cryptic sites other than the usual P1 or P2 initiation start site (promoter shift) [15,20,24]. Increased transcriptional activity is observed in breakpoints within exon 1 and intron 1 (Class I) than when it occurs within the most common breakpoint, 5’ from MYC exon 1 (Class II) [15]. Long-range cis-acting enhancers regulate *MYC* expression through chromatin looping bringing the enhancers in close proximity to *MYC*[25,26], or through increased distal enhancer activity utilizing preexisting loops [27]. Multiple genetic variants and SNPs, located in 1.5 Mb “gene desert” regions 1, 2 and 3, up to 600 Kb upstream of *MYC*, are associated with increased susceptibility to prostate, colorectal, bladder, breast cancer, or chronic lymphocytic leukemia [26-28]. Although reporter expression studies revealed that long-range enhancers and other regulatory elements regulate *MYC* transcription, the clinical significance of *MYC* rearrangements upstream of *MYC* remain unclear and a subject of burgeoning field of investigation [4].

To date, there are only very few reported cases of CLL with apparently balanced t(8;14) and atypical rearrangements [6,8], none of which exhibits abnormal FISH signal patterns similar to what we detected in our patient. These abnormal patterns include: cryptic deletion on 8q24.1 including *MYC*[6,8], gain of an extra copy of *MYC (+MYC*) [4,5,29], or deletion of IGH@, usually 5’ [3,4,6,30,31]. The prognosis for these cases is also variable, from indolent to transformed into a more aggressive course.

Here we report a CLL case with complex cytogenetic findings of deletions of 11q and 13q, in addition to the apparently balanced t(8;14). We also present an undocumented atypical complex rearrangements involving 3’-*IGH@* and at least 400 Kb upstream of 5’-*MYC*, unreported complex atypical rearrangements of *IGH@* and *MYC* loci that did not result in IGH-*MYC* fusion and no subsequent MYC expression.

### Clinical report

**Figure 1:**
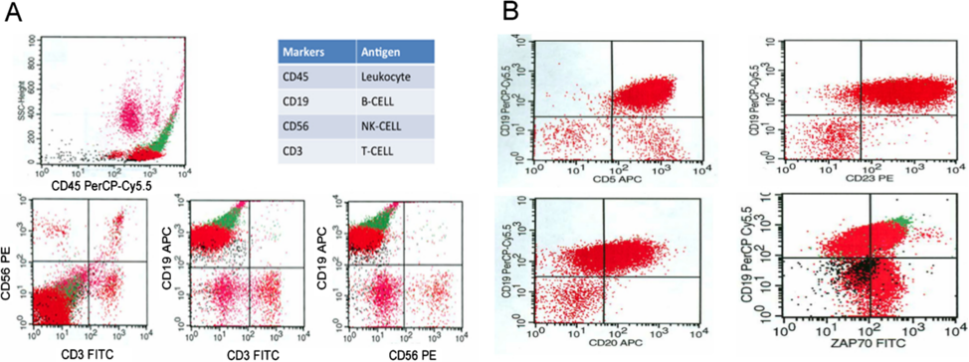
**Flow Cytometry Analysis in Peripheral Blood. A**. The lymphoid gated population constitutes 87% of total cells, and consists of 2% T cells, 70% B cells, and <2% NK cells. **B**. Coexpression of CD19 B-cell antigen with CD5, CD23, CD20, and ZAP70, findings characteristic of CLL.

Our patient is a 67 year-old Hispanic male with a medical past history of an end-stage kidney disease of uncertain etiology. His white blood cell count (WBC) was elevated and measured at 23.15 × 10^3^ per μL. Peripheral blood smear showed marked lymphocytosis with numerous atypical lymphoid cells including prolymphocytes, smudge cells, normocytic normochromic anemia and thrombocytopenia. The lymphoid-gated population constituted 87% of total cells, and consisted of 2% T cells, 70% B cells, and <2% NK cells (Figure 1A). Flow cytometry showed co-expression of B-cell antigen (CD19) with CD5, CD23, CD20, and ZAP70 expression. These results were suggestive of CLL (Figure 1B).

## Results

**Figure 2:**
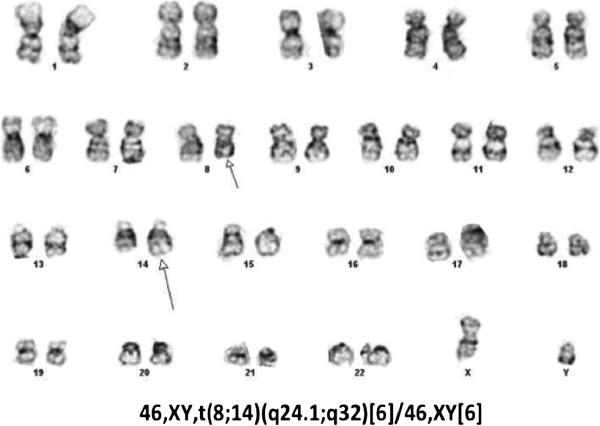
G-Banding karyotype of our patient’s peripheral blood reveals an apparently balanced t(8;14)(q24.1;q32).

A complete chromosome analysis was not possible due to low mitotic index. G-banded karyotype analysis of available metaphase cells revealed an abnormal male karyotype with an apparently balanced t(8;14)(q24.1;q32) seen in 50% (6/12) of total cells examined (Figure 2).

**Figure 3:**
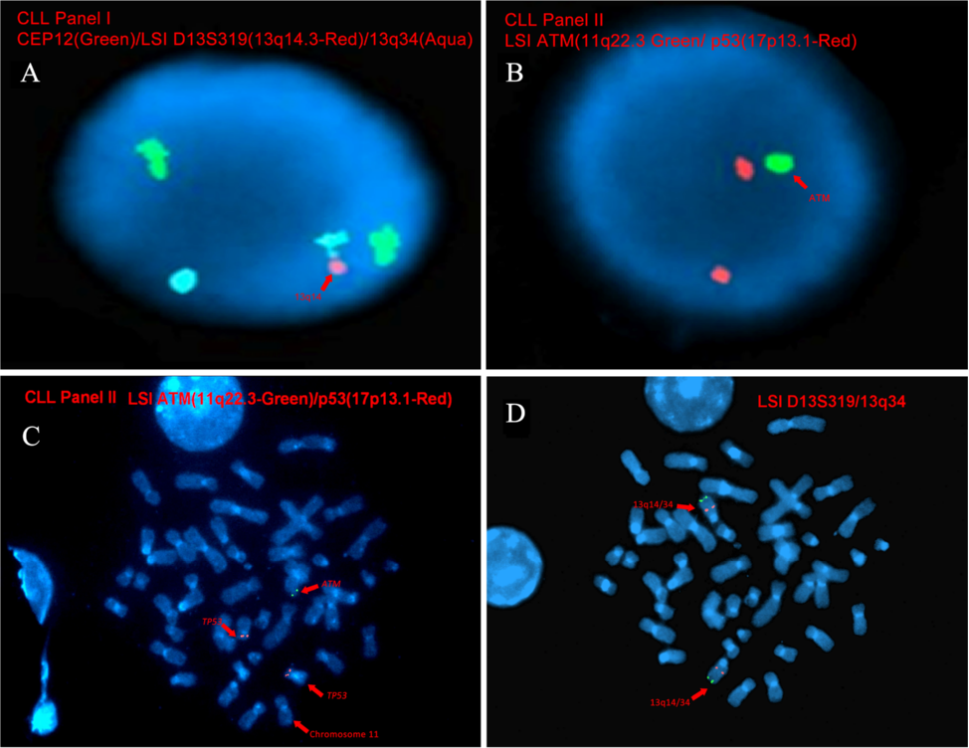
**Interphase and Sequential Metaphase FISH in our Patient’s Peripheral Blood using our CLL Panel. A**-**B**: Interphase Nuclei FISH detects: (**A**) deletion of 13q14.3 (D13S319) (2G1R2A), and (**B**) deletion of 11q22.3 (2G1R). The signal patterns for chromosome 12 centromere, 13q34 and 17p13.1 are normal. **C**-**D**: Sequential FISH on the metaphase cell with t(8;14) using: (**A**) *ATM *(green)/*TP53 *(red) specific probes reveals a loss of one *ATM* signal (2G1R), and (**B**) with 13q14.3/13q34 specific probes did not reveal a deletion of 13q14.3 in this cell. These findings indicate two separate abnormal clones, one with t(8;14) and 11q deletion, and another with 13q14.3 deletion.

**Figure 4:**
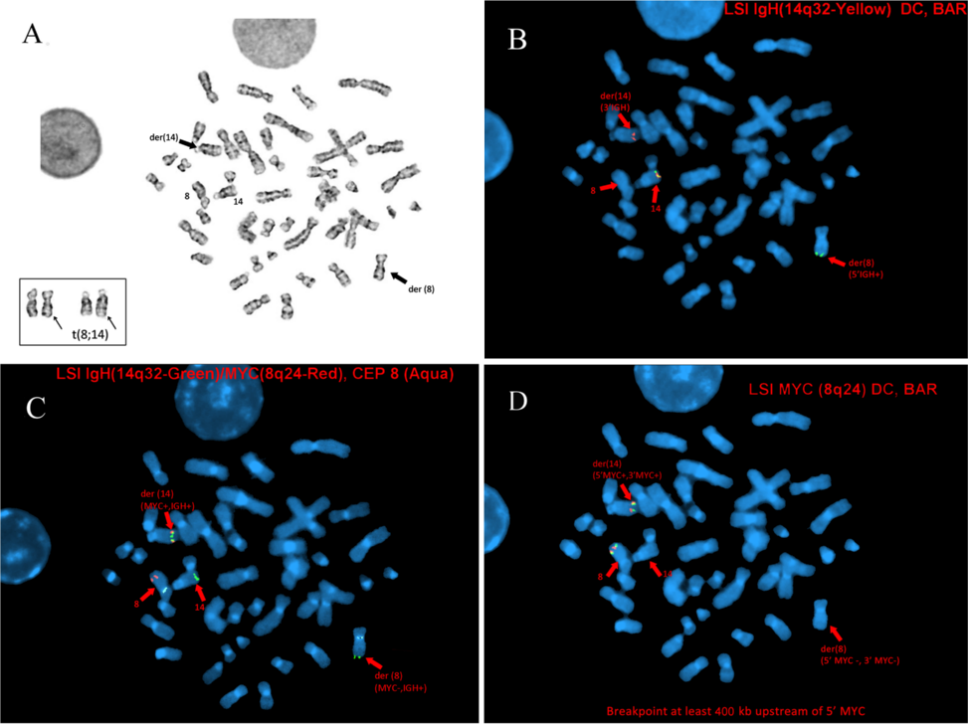
**Further Examination of the Rearrangement Involving the *MYC *and *IGH@ *Loci using Sequential Metaphase FISH in our Patient’s Peripheral Blood. A**: G-Banding metaphase spread showing an apparently balanced t(8;14)(q24.1;q32). **B**: The IGH@ break apart probe reveals splitting of signals (1Y1G1R) indicative of rearrangement, with 5’-*IGH@ *relocated to der 8q and 3’-*IGH@ *retained on der 14q. **C**: The IGH-MYC fusion probe shows 1Y2G1R2A, fusion of MYC-IGH der 14q32 (yellow), and deletion of MYC on der 8q24.1 (green, no red). It also shows normal signal patterns for the other chromosome 8 (aqua for centromere and red for MYC), and chromosome 14 (green). **D**: The MYC break apart probe detects 2Y signals, one on normal chromosome 8 and the other is removed from der 8q24.1 and relocated to der 14q32. These findings suggest that there is neither splitting of signals, or a deletion in between the red and green signals.

Interphase FISH studies did not reveal IGH@-CCND1 rearrangement, but instead, an extra copy of IGH@-specific signal in 30.3% (91/300) of nuclei examined (data not shown). In addition, deletions of the 13q14.3 (D13S319) (Figure 3A) and 11q22.3 (*ATM*) (Figure 3B) in 8% (24/300) and 78% (294/300) of cells were also observed, respectively. Neither deletion of 17p13.1 (*TP53*) nor trisomy 12 was detected (data not shown). Sequential GPG-metaphase FISH studies were performed on the same chromosome metaphase spread to determine the clonality of the structural abnormalities seen in our patient. Results showed that two different clones exist in the peripheral blood of our patient: one with deletion 13q14.3 (seen only in interphase nuclei in our study), and another with concurrent deletion 11q (Figure 3C) and t(8;14) (Figure 4D).

**Figure 5:**
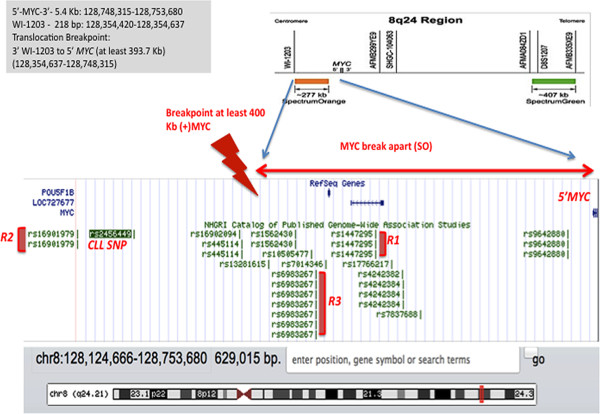
***In Silico *Mapping of the Translocation Breakpoint Upstream of 5’-MYC-3’ Reveals a Class III Breakpoint and a Region that Contains SNPs associated with Susceptibility Risk Loci for different types of cancer including CLL, prostate, colorectal, bladder, or breast cancer.** The farthest probe upstream of 5’ of *MYC* is the Spectrum Orange of the MYC break apart probe (upper panel) (adopted from Abbott Vysis website for FISH probes). We plotted the base coordinates for WI-1203 STS marker and the 5’ of MYC (see inlet) into the UCSC Genome Browser (hg19) and determine the distance, RefSeq genes (*POUF51B* and *LOC727677*), STS markers and SNPs within this interval. The translocation breakpoint (with lightning icon) is centromeric of WI-1203 and about ~400 Kb upstream of 5’-MYC, a Class III breakpoint. We extended the breakpoint further upstream to show ~629 Kb region containing SNPs in different regions of the interval (R1, R2, R3 and CLL) that confer susceptibility risks for cancer [26,28].

Further sequential FISH studies on 10 metaphase nuclei using the IGH@ break apart probe showed splitting or rearrangement (1Y1G1R), with the 5’-IGH@ (green) translocated on chromosome 8q24.1 and the 3’-IGH@ (red) retained on 14q (Figure 4A,B) in all cells examined. The IGH@-MYC fusion (Figure 4C) and MYC break apart (Figure 4D) probes revealed atypical abnormal signal patterns in all 10 cells examined on derivative 8q24.1: one green (5’-IGH@) and no red (deletion at least 400 Kb upstream of 5’-*MYC*-3’); and, on derivative 14q32: one yellow (relocation of 5’-*MYC*-3’ and its flanking regions adjacent to 3’-IGH@). The estimated location of the translocation breakpoint upstream of 5’-MYC was determined by *in silico* mapping (Figure 5) by determining the base pair coordinates in the UCSC Genome Browser (hg19) of the STS markers mapped upstream of 5’-*MYC*-3’ (Abbott Vysis FISH probes website). We based our calculations on the Spectrum Orange of the MYC break apart probe, the farthest probe from 5’ of MYC (as compared to the MYC probe in the IGH-MYC fusion probe). The estimated distance of the translocation breakpoint (STS marker WI-1302) from 5’of *MYC* is at least 400 Kb (bp 128,354,420-128,747,680). This interval includes two RefSeq genes: *POU5F1B* (POU class 5 homeobox 1B), an intronless gene that encodes for a transcription factor (1.6 Mb; bp 128,427,857-128,429,441) and a gene with no known function, *LOC727677* (38.8 Kb; bp 128,455,595-128,494,384). It also includes the SNPs implicated in several cancer types, rs1447295 (Region 1), rs16901979 (Region 2) and rs6983267 (Region 3) [26] and CLL SNP rs2456449 [28].

SNP CMA refined the 11q22.3 deletion breakpoints detected by FISH. Results showed a 7.1 Mb heterozygous copy number loss at chromosome 11q22.3-q23.3, arr 11q22.3q23.3(107,888,769-115,016,307)x1 (data not shown). It deleted 62 RefSeq genes including *ATM* (ataxia telangiectasia mutated), a gene that encodes for a cell cycle checkpoint phosphorylating kinase that functions for regulating proteins for tumor suppression, checkpoint, DNA repair and maintenance of genome stability [32]. In addition, a 455 Kb heterozygous copy number loss on 14q32.33 was also detected; arr 14q32.33(106,530,533-106,985,955)x1, deleting two gene fragments or non-protein coding genes of no known function, LINC00226 and LINC00221 (data not shown) [32]. CMA did not detect a microdeletion within or surrounding the *MYC* locus despite its removal from der 8q24.1 locus. This suggests that there was no net gain or loss despite the unbalanced rearrangements detected by FISH. In a lesser extent, a 61 Kb gain on 8q24.12 was detected, but found to be unreportable with further *in silico* investigations. There were no clinically relevant gains or losses detected on chromosomes 12, 13 and 17.

According to the ISCN [33], the overall findings from karyotype, FISH and CMA can be described as: 46,XY,t(8;14)(q24.1;q32).ish der(8)t(8;14)(q24.1;q32)del(8)(q24.1q24.1)(MYC-,5’IGH@+),der(14)t(8;14)(MYC+, 3’IGH@+),del 11q22.3q22.3

(ATM-),13q14.3q14.3(D13S319x2)[10].nuc ish(ATMx1,TP53x2)[294/300], (D13S319x1,13q34x2)[24/300], (CCND1x2,IGH@x3)[91/300].arr 11q22.3q23.3(107,888,769-115,016,307)x1,14q32.33(106,530,533-106,985,955)x1.

As mentioned above, deletion 13q14.3 and deletion 11q22.3 with t(8;14), detected by interphase and sequential metaphase FISH studies, are found as two different abnormal clones, indicative of mosaicism. CMA failed to detect gains or losses on 13q, since it only accounts for 8% of the total cell population, a number way below the detection limit (10-30%) of either SNP or BAC microarrays [30].

**Figure 6:**
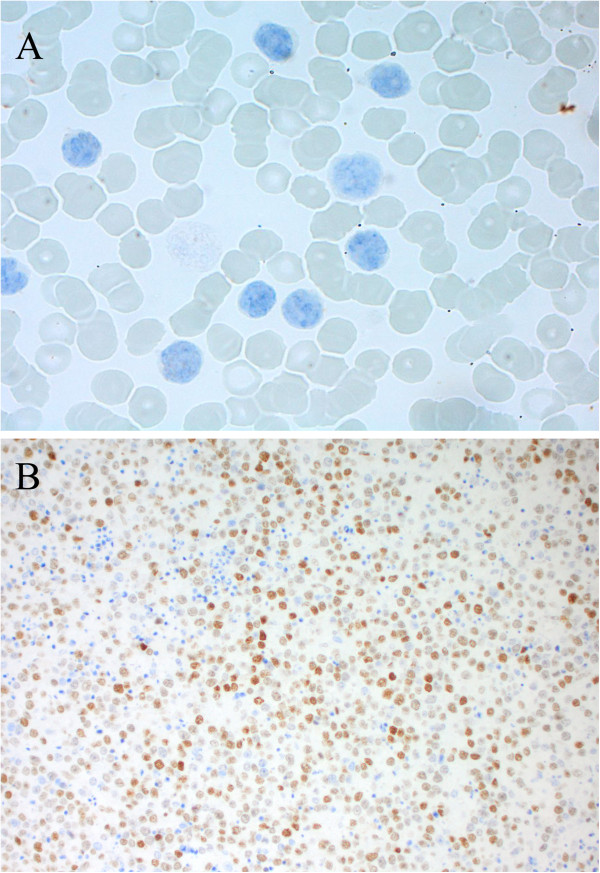
**Immunohistochemistry using human anti-*MYC *antibody. A**. Positive *MYC *expression on a positive control. **B**. Negative *MYC *expression in our patient’s blood smear.

Immunohistochemistry studies using specific *MYC* antibodies did not detect any staining in our patient’s sample, suggestive of absence of *MYC* activation (Figure 6A). A strong positive staining for *MYC* was detected for the positive control sample (Figure 6B).

## Discussion

Our patient’s clinical, morphological and immunophenotypic features are consistent with the diagnosis of CLL. Although complex cytogenetic findings including t(8;14) usually confers poor prognosis in CLL, a consistent genotype and phenotype correlation remains an unresolved issue. Our patient’s case exhibits an unreported rearrangement involving *IGH@* and *MYC* loci with absence of *MYC* expression.

In our patient, the FISH signal patterns detected are unique from those previously reported in CLL cases with atypical rearrangements and an apparently balanced t(8;14). These include a cryptic deletion on 8q24.1 including *MYC*[6,8], gain of an extra copy of *MYC (+MYC*) [4,5,29], or deletion of IGH@, usually 5’ [3,4,6,30,31]. Although a deletion of the *MYC*-specific signal on der 8q24.1 locus was also observed in our patient using IGH-MYC fusion probe (1Y2G1R), it is not identical to the reported deletion by Reddy et al. in 2006 [6,8]. The deletion reported on here did not show splitting of signals and no concomitant deletion of a 1.6 Mb segment including the *MYC* locus. Instead, it showed two unsplit MYC probes (yellow) on the normal chromosome 8 and on der 14q32. We interpreted these findings as an atypical rearrangement never reported elsewhere, with the 5’-*MYC*-3’ removed from the 8q24.1 locus at a breakpoint at least 400 Kb upstream of its 5’ region. We also showed that this deleted region is relocated to the 14q32 locus and apposed to the 3’-IGH@ locus. Neither gain of *MYC* nor deletion of the *5’-IGH@* locus was observed by FISH or CMA in our case. We have exhaustively searched the available literature and did not find any cases similar to the signal patterns reported on here.

To the best of our knowledge, expression levels of *MYC* and its correlation to disease progression have not been established in CLL with t(8;14), with or without *MYC* translocations [4,6,7]. *MYC* expression is generally at low levels in CLL [23], and similar in groups with either bad or good prognosis [34]. Increased expression even without *MYC* rearrangement has also been described in CLL with malignant Richter transformation and other higher risk cases for CLL progression [10]. Although, high levels of *MYC* are expressed as a result of the t(8;14) and its variant translocations in Burkitt’s lymphoma and in some other B-cell malignancies including DLBCL and plasma cell myeloma, these translocations may not necessarily lead to increased expression of *MYC* in CLL [4,6,7]. These variable findings of *MYC* expression are most likely dependent on specific disruptions of regulatory regions, or characteristic genomic translocation breakpoints either at the *MYC* or *IGH@* locus.

The typical *MYC-IGH* fusion at der 14q32 expresses the *MYC* -deregulatting product, while the reciprocal *IGH-MYC* fusion at 8q24.1 locus is transcriptionally silent [14,35]. Despite the typical juxtaposition, overall *MYC* expression in some CLL cases remains within the normal range [20], or overexpressed through processes other than translocations [9]. It has been reported that the location of the genomic breakpoint influences *MYC* expression, with highest level when involving Class I breakpoints [15,24]. The absence of Myc expression in our patient is most likely due to the atypical *MYC-IGH* fusion on der 14q32, with a Class III breakpoint (at least 400 Kb upstream of *MYC*) [16].

The previously reported “gene desert” region upstream of *MYC* extends up to about 629 Kb [26] and includes genes and SNPs. Genome-wide association studies (GWAS) have shown the gene *POU5F1B* and several genetic variants or SNPs (Regions R1, R2, R3) (Figure 5) that are risk factors for various cancers including CLL exist in this region [26-28,36-38]. The strongest evidence for risk or genetic susceptibility in CLL or monoclonal B-cell lymphocytosis is rs2456449 (8q24.21) [28,38]. In our patient, the breakpoint that we suggested (at least 400 Kb) is within this interval and includes *POU5F1B*, and SNPs R1 and R2. *POU5F1B* is one of the two RefSeq genes within the breakpoint on 8q24.1 and 5’-*MYC*, is the most adjacent. Although it is not yet well studied, few reports described it as a pseudogene or a gene that encodes for a weak transcription factor that may play a critical role in stem cell pluripotency, eye development and carcinogenesis [36,37,39]. At the present time, there are no reports of a specific fusion involving 5’-*POU5F1B* and 3’-regulatory region of *IGH@.* It is possible that the breakpoint in our patient is further upstream, however, the paucity of available cells made it impossible for further characterization. In Figure 5, we extended the suggested breakpoint further upstream, from ~400 Kb to ~600 Kb, to include the farthest reported cancer-associated SNP (Region 2: rs16901979) and CLL SNP (rs2456449). To date, the genotype phenotype correlation underlying these associations remains unclear. However, it has been suggested by reported expression studies that *MYC* expression is influenced by such SNPs variants by altering its transcription regulation and amplification [40]. Despite such plethora of reports, replication of these findings and elucidation of its physiologic function and clinical significance remain an area of thorough investigation. Further *in vivo* and *in vitro* functional studies are needed to show consistent association of risk allele status and *MYC* expression levels.

On the other hand, transcription at the *IGH@* locus is controlled by enhancers elements spread out as wide as 2.5 Mb of the locus [12], and it contains regulatory elements necessary not only for *MYC* activation but also the promotion of translocation [13]. CMA detected a 455 Kb copy number loss on chromosome band 14q32.2, not detected by FISH since the probe used was outside of this region. It is still a possibility, that the deletion in our patient may have removed some of the regulatory elements within this interval somehow affecting the regulation of Myc expression. No regulatory elements or high conservation data was seen in the UCSC Genome Browser. This microdeletion has been reported in other CMA studies of CLL patients using BAC-based array CGH, with some of the cases exhibiting the same findings as ours, i.e. with no *IGH@* deletion by FISH [30]. It is still unclear whether this microdeletion is a polymorphic feature of this locus and represents a region of frequent mutation and recombination, or it exhibits some susceptibility risks for CLL [3,24,31,41].

About 20% of patients with CLL show *ATM* deletion, an anomaly also seen in almost all cancer, and is usually associated with an adverse outcome [1,4,31]. The collaboration of *ATM* and *MYC* in normal cell proliferation via an ATM-dependent pathway is well established. When deleted, *ATM* loses its protective checkpoint function leading to MYC-induced oncogenesis [4,42]. This indicates that *MYC* alone is not capable of transforming lymphoid cells into neoplasia [4]. The *ATM* deletion and removal and relocation of *MYC* observed in our patient may explain the lymphomagenesis, but not necessarily the absence of Myc expression.

Given the limitations of this case report, we suggest that comprehensive retrospective studies in CLL patients should be performed to characterize the suggested ~400 Kb breakpoint and the region further upstream by sequential metaphase BAC FISH mapping since CMA does not detect the removal and relocation of an intact MYC locus. It is also possible that the absence of Myc expression is a false negative result given the specificity of immunostaining which is below 100%, and about 17% of cases may be overlooked for *MYC* rearrangements using this technique [43]. A more accurate quantitative approach such as RT-qPCR is recommended. Since variability in *MYC* breakpoints could still result in similar *MYC* expression [44], possibly due to flexible DNA looping [43,45], reporter expression studies are needed to better understand the clinical impact and significance of long distance deregulation in in loci with atypical *MYC* rearrangement.

This paper presents an unreported atypical rearrangement involving the *IGH@* and *MYC* loci detected by FISH, adding to the burgeoning cytogenetic data on CLL patients with atypical t(8;14). It also highlights the Class III translocation breakpoint upstream of *MYC*, including the cancer and CLL-associated SNPs within the interval. This report also provides important and promising findings for further studies correlating Myc expression with a specific type of genomic translocation breakpoint or copy number variants in CLL and in other B-cell disorders. Lastly, overall findings in our report highlight the utility of karyotype analysis, interphase and sequential metaphase FISH studies, CMA, and other molecular tools in approaching the diagnosis and prognosis of CLL in a more comprehensive manner.

## Materials and methods

Conventional GPG-banded chromosomal analysis was performed on peripheral blood lymphocytes that were cultured for 48 and 72 hours with and without pokeweed mitogen stimulation, following standard cytogenetics protocols. The karyotypes were described according to the ISCN 2009 nomenclature [33].

Initial FISH studies were performed on interphase cells using CLL panel probes (Abbott Molecular, Des Plaines, Illinois) specific for centromere 12, IGH@ break apart or IGH@-CCND1 fusion, and chromosome loci 13q14.3 (D13S319)/13q34, 11q22.3 (*ATM*) and 17p13.1 (*TP53*). Sequential GPG-metaphase FISH studies were performed using IGH-MYC fusion (with centromere 8-specific probe), and break apart probes for the IGH@ and MYC loci.

Chromosome microarray analysis was performed on DNA sample from 48-hr culture of peripheral blood lymphocytes. DNA was extracted from Carnoy’s fixed pellet cells Qiagen DNA extraction kit (Valencia, CA). DNA concentration and quality was checked using Nanodrop (Life Technologies, Carlsbad, CA) and gel electrophoresis, respectively. Whole genome chromosome SNP microarray was performed to assess for imbalances (i.e. gain or losses) in the genomic DNA sample tested. The assay compared the patient’s DNA to a reference set from 380 normal controls (284 HapMap and 96 Affymetrix reference), using the Genome-Wide SNP Array CytoScan HD. This array platform contains 2.6 million markers for Copy Number Variant detection (Affymetrix, Inc.), which 750,000 are genotype SNPs and 1.9 million are non–polymorphic probes, for the whole genome coverage. The analysis was performed using the chromosome analysis suite (ChAS), version CytoB-N1.2.2.271(r4615). Oligonucleotide probe information is based on the 37 build of the Human Genome (UCSC Genome Browser, http://genome.ucsc.edu/cgi-bin/hgGateway, hg19, February 2009).

FFPE sections (4 μm thick) were stained for Myc using rabbit monoclonal human anti-Myc antibody (catalog #1472-1, Epitomics, Inc., Burlingame, CA, USA). Heat-induced epitope retrieval was accomplished by using ER1 for 20 min. Endogenous peroxidase was blocked using hydrogen peroxide. The slide was incubated in the primary antibody Myc for 30 min, followed by incubation in a post-primary 3,3-diaminobenzidine for 10 min, polymer 3,3-diaminobenzidine for 10 min, and chromogen 3,3-diaminobenzidine for 10 min. Subsequently, slide was incubated with post-primary alkaline phosphatase for 20 min, polymer alkaline phosphatase for 30 min, and fast red for 20 min. The nuclei were counterstained with hematoxylin and the slide was then dehydrated, cleared in xylene, and coverslipped. Appropriate positive controls were used.

## Competing interests

The authors declare they have no competing interests.

## Authors’ contributions

IA performed metaphase studies; gathered data for karyotype, interphase and metaphase FISH studies; analyzed, interpreted and wrote the cytogenetic report; analyzed and interpreted chromosome microarray data; and did necessary revisions in the manuscript as requested by the reviewers. PHB analyzed the molecular data, correlated clinical findings, and wrote the initial draft of the paper. BS provided the flow cytometry and immunohistochemistry data. SK reviewed the microarray data and paper draft. CT analyzed and reviewed all the data and drafted the paper. All authors read and approved the final manuscript.
